# Gene silencing versus protein stabilization in transthyretin amyloid cardiomyopathy: contextualizing the HELIOS-B results and the road to precision agent selection

**DOI:** 10.1097/MS9.0000000000005215

**Published:** 2026-06-09

**Authors:** Sarum Ali Khan, Taimoor Ali, Muhammad Sajjad Ul Haque, Muhammad Adnan Qamar

**Affiliations:** a Wah Medical College, Wah Cantt, Pakistan; b Spinghar Medical University, Kabul, Afghanistan

**Keywords:** eplontersen, gene silencing, HELIOS-B, precision cardiology, RNAi, tafamidis, transthyretin amyloid cardiomyopathy, vutrisiran

## Abstract

Transthyretin amyloid cardiomyopathy (ATTR-CM) now has three approved disease-modifying agents: tafamidis, acoramidis, and vutrisiran, representing two mechanistically distinct classes: TTR tetramer stabilizers and RNA interference (RNAi) gene silencers. The phase 3 HELIOS-B trial, published in the New England Journal of Medicine, demonstrated that vutrisiran, a subcutaneous RNAi therapeutic, reduced the composite of all-cause mortality and recurrent cardiovascular events compared with placebo (hazard ratio 0.72; *P* = 0.01) in 655 patients with ATTR-CM over 33–36 months. These results supported the March 2025 FDA approval of vutrisiran for this indication. This letter contextualizes the mechanistic shift from stabilization to silencing, discusses the limitations of the HELIOS-B trial design, and examines the implications of an expanding therapeutic landscape for which patient-level selection criteria remain undefined. The ongoing CARDIO-TTRansform trial of the antisense oligonucleotide eplontersen will provide further comparative data, but head-to-head evidence across existing approved agents is absent. The question of how to sequence or select between mechanistically distinct agents for individual patients represents the central unresolved issue in ATTR-CM management.

*Dear Editor,*

We read with interest the HELIOS-B trial by Fontana *et al*, published in the *New England Journal of Medicine*, which evaluated vutrisiran in 655 patients with transthyretin amyloid cardiomyopathy (ATTR-CM) over a double-blind period of 33–36 months[1]. Vutrisiran, a subcutaneous small interfering RNA administered once every 3 months, reduces hepatic synthesis of wild-type and variant transthyretin (TTR) by approximately 80%, targeting production at its source rather than stabilizing the already-formed tetramer[1]. This distinction matters: TTR stabilizers bind the tetramer to prevent monomer dissociation, leaving residual circulating TTR intact, whereas RNA interference agents reduce total TTR production by degrading mRNA hepatically. In HELIOS-B, treatment with vutrisiran led to a lower risk of the composite of all-cause mortality and recurrent cardiovascular events compared with placebo (hazard ratio 0.72; 95% CI 0.56–0.93; *P* = 0.01) in the overall population, and a lower risk of all-cause mortality alone at 42 months (hazard ratio 0.65; 95% CI 0.46–0.90; *P* = 0.01)[1].

These results require contextualized interpretation. Approximately 41% of participants in HELIOS-B were receiving concomitant tafamidis at baseline, complicating attribution of effect to vutrisiran monotherapy; prespecified subgroup analyses in the monotherapy population (hazard ratio 0.67; 95% CI 0.49–0.93) and those on background tafamidis showed directionally consistent but quantitatively heterogeneous results, and HELIOS-B was not powered to establish statistical significance within the tafamidis subgroup[1]. Additionally, prespecified analyses suggested that younger patients, those with wild-type disease, lower baseline NT-proBNP, and no concomitant tafamidis use derived numerically greater benefit[2]. The trial enrolled a predominantly male (92%) and largely wild-type ATTR-CM population, limiting generalizability to hereditary variant disease, including the Val122Ile variant prevalent in populations of West African ancestry, among whom ATTR-CM is substantially underdiagnosed[3].

The expansion of ATTR-CM therapeutics to three approved agents raises the practical question of selection and sequencing, for which direct comparative evidence is absent. No head-to-head trial has compared vutrisiran against tafamidis or acoramidis in ATTR-CM. Cross-trial comparisons are confounded by differences in populations and endpoints; the ATTR-ACT trial enrolled a sicker population with higher event rates than HELIOS-B, limiting direct inference[3]. Eplontersen, a ligand-conjugated antisense oligonucleotide that also silences hepatic TTR production, is undergoing evaluation in the phase 3 CARDIO-TTRansform trial (NCT04136171), which is enrolling over 1400 patients with ATTR-CM with or without background tafamidis. The trial is designed to address some of the combination therapy questions left unanswered by HELIOS-B[4]. Until these data are available, clinicians face agent selection without comparative evidence, particularly for patients with concurrent polyneuropathy, whose phenotypic overlap between cardiac and neurological ATTR remains undertreated when agents are chosen for a single-system indication[3].

Several open questions will shape ATTR-CM management. Whether gene silencing and tetramer stabilization are additive, redundant, or potentially antagonistic when combined has not been tested in randomized conditions; HELIOS-B was not designed to answer this, and the CARDIO-TTRansform trial will be the first to provide interpretable data on this question[4]. The long-term safety of sustained TTR suppression also requires monitoring, given TTR’s known role as a retinol-binding protein. Vitamin A supplementation is routinely co-administered with eplontersen for this reason, and long-term TTR suppression monitoring data for vutrisiran beyond 42 months remain unavailable[1]. The diagnostic gap in ATTR-CM, especially in regions where technetium scintigraphy and genetic testing are inaccessible, means that the availability of approved therapies is not matched by the capacity to identify candidates for them in many healthcare settings[3].

The HELIOS-B trial provides phase 3 evidence that vutrisiran reduces mortality and cardiovascular events in ATTR-CM, representing a mechanistically distinct addition to the treatment landscape[1].

Its limitations, including heterogeneous tafamidis co-administration, predominance of wild-type disease, and absence of head-to-head comparators, mean that agent selection currently rests on indirect inference. Prospective trials addressing sequencing, combination, and subgroup-specific efficacy will be essential to translate the expanding therapeutic options into individualized care.

## Data Availability

All data used are accessible.
